# A Hölder-type inequality for the Hausdorff distance between Lagrangians

**DOI:** 10.1007/s11784-025-01177-4

**Published:** 2025-03-20

**Authors:** Jean-Philippe Chassé, Rémi Leclercq

**Affiliations:** 1 D-MATH, ETH Zürich, Rämistrasse 101, 8092 Zurich, Switzerland; 2 Laboratoire de mathématiques d’Orsay, CNRS, Université Paris-Saclay, 91405 Orsay, France

**Keywords:** Symplectic geometry, Lagrangian submanifolds, Hausdorff distance, spectral and Hofer–Chekanov distances, Primary 53D12, Secondary 53C17

## Abstract

We prove a Hölder-type inequality (in the spirit of Joksimović and Seyfaddini in Int Math Res Not IMRN 8:6303–6324, 2024) for the Hausdorff distance between Lagrangians with respect to the Lagrangian spectral distance or the Hofer–Chekanov distance. This inequality is established via methods developed by the first author (Chassé in Int J Math 34(5):2350024, 2023; Chassé in Differ Geom Appl 94:Paper No. 102123, 22, 2024) to understand the symplectic geometry of certain collections of Lagrangians under metric constraints.

## Introduction

Let $(M,\omega )$ be a symplectic manifold with an $\omega$-compatible almost complex structure *J*. If *M* is noncompact, we assume that *J* is convex at infinity. We equip *M* with the Riemannian metric $g=g_J=\omega (\cdot ,J\cdot )$—we may assume it is complete and geometrically bounded (cf. [5]).

### Main result

On one hand, Joksimović and Seyfaddini [8] proved a Hölder-type inequality for the $C^{0}$ distance on Hamiltonian diffeomorphism groups and deduced interesting applications to Anosov–Katok pseudo-rotations.

Namely, the inequality is the following:

1.1 $$\begin{aligned} d_{C^0}(\mathbbm {1},\varphi )\le C\sqrt{\gamma (\varphi )} \, \Vert \textrm{d}\varphi \Vert , \end{aligned}$$

where $\Vert \textrm{d}\varphi \Vert :=\sup \{ |\textrm{d}\varphi _x|^\textrm{op} \,|\, x \in M \}$ and $|\cdot |^{\textrm{op}}$ is the operator norm $T_xM\rightarrow T_xM$ induced by the Riemannian metric. This inequality holds for any Hamiltonian diffeomorphism $\varphi$ of a closed symplectic manifold for which one can define Floer spectral invariants. These invariants and their properties are reviewed in Sect. 2. They induce, on the Hamiltonian diffeomorphism group, the *spectral pseudonorm* $\gamma$ which appears in the inequality. The constant *C* only depends on the choice of a Riemannian metric on the ambient manifold.

On the other hand, the first author [2, 3] initiated the study of the symplectic geometry of certain sets of Lagrangians under (Riemannian) metric constraints, such as the Hofer geometry of Hamiltonian isotopic Lagrangians with uniformly bounded curvature. Hölder inequalities on such sets between the Hausdorff distance and a large class of metrics were also obtained. This class of metrics contains, in particular, the *Lagrangian spectral distance*, also defined via spectral invariants and denoted $\gamma$ as well. This version of the spectral distance was defined for weakly exact Lagrangians in [11] and for monotone Lagrangians with nonvanishing fundamental class in [10].

The upshot of this note is a Hölder-type inequality for the Hausdorff distance $\delta _H$ between Lagrangians in the spirit of Joksimović and Seyfaddini’s inequality, whose proof is based on the methods of [2].

#### Theorem 1.1

Let *L* and $L'$ be Hamiltonian isotopic, closed, connected Lagrangian submanifolds of *M*. Suppose that *L*—and thus $L'$—is either weakly exact or monotone with $N_{L} \ge 2$ and has nonvanishing quantum homology. Let $\psi$ be a symplectomorphism of *M* such that $\psi (L)=L'$. There exist constants $\delta =\delta (M,J,L)>0$ and $C=C(M,J,L)>0$ such that whenever $\gamma (L,L')< \delta$, then

1.2 $$\begin{aligned} \delta _H(L,L')\le C\sqrt{\gamma (L,L')}\ \Vert \textrm{d}\psi \Vert . \end{aligned}$$

Furthermore, when *M* is compact, we may take $\delta =+\infty$.

This obviously yields the nondegeneracy of $\gamma$.

#### Corollary 1.2

Let *L* be a Lagrangian as in Theorem 1.1. Then, the Lagrangian pseudodistance $\gamma$ is nondegenerate on the set of Lagrangians which are Hamiltonian isotopic to *L*.

Thus, this provides a third proof of this result, after Kawasaki’s proof [9] via Poisson bracket invariants *à la* Polterovich and Rosen [14] and Kislev and Shelukhin’s proof [10] via energy-capacity inequalities. However, through the use of the methods of [2] in Sect. 3 or with the more direct approach of the alternative proof in Sect. 4.1, the proof does ultimately relies on the same existence result for certain *J*-holomorphic curves as Kislev and Shelukhin [10]. The innovation here is how we estimate the area of those *J*-holomorphic curves.

#### Remark 1.3

Ultimately, the proof of Theorem 1.1 relies on [3, Lemma 5], which is stated for Chekanov-type *metrics*. However, the only requirement there is that the associated families have a nice enough intersection, which is automatically satisfied for the spectral pseudo-metric because, in that case, said families are empty. Hence, we do not require *a priori* $\gamma$ to be non-degenerate.

Before describing in more detail how this result relates to the aforementioned previous works, let us make several quick remarks.

#### Remark 1.4

(Hofer’s geometry) It is well known that $\gamma$ is bounded from above by the Hofer–Chekanov distance—see the properties of the spectral distance in Sect. 2. Hence, Theorem 1.1 also holds when $\gamma$ is replaced by the Hofer–Chekanov distance.

#### Remark 1.5

(Symplectomorphisms) Let us emphasize the fact that Theorem 1.1 holds for any—even noncompactly-supported—*symplectomorphism* $\psi$; *L* and $L'$ are required to be Hamiltonian diffeomorphic only for $\gamma (L,L')$ to be defined.

This is a nontrivial improvement, as one can see with a simple example. For example, take $f(x)=\sin (2\pi x)$ and consider $L=\operatorname {graph}(f')$ and $L'=\operatorname {graph}(-f')$ in $T^*S^1$. If we equip $T^*S^1$ with the flat metric, then the symplectomorphism $\psi (x,y)=(-x,-y)$ sends *L* to $L'$ and is also an isometry, i.e. $\Vert \textrm{d}\psi \Vert =1$. However, the only nontrivial isometries of the flat cylinder that are in the connected component of the identity (in $\operatorname {Diff}(T^*S^1)$) are translations, none of which are Hamiltonian diffeomorphisms. Therefore, every Hamiltonian diffeomorphism $\varphi$ sending *L* to $L'$ must have $\Vert \textrm{d}\varphi \Vert >1$.

#### Remark 1.6

(Variant with the norm of the inverse diffeomorphism) When *M* is compact, there is also an inequality involving $\Vert \textrm{d}\psi ^{-1}\Vert$:

1.3 $$\begin{aligned} \delta _H(L,L')\le C\sqrt{\gamma (L,L')}\ \Vert \textrm{d}\psi ^{-1}\Vert ^2. \end{aligned}$$

This variant of inequality (1.2) will be proved in Sect. 4.2.

### Main techniques and relations to previous work

Theorem 1.1 is a specialization of the first author’s inequality from [2], which we now recall. For any metric *D* in a large class of metrics, said of Chekanov type and which includes $\gamma$, if $D(L,L')<\delta =\delta (g,g|_L,g|_{L'})$, then

1.4 $$\begin{aligned} \delta _H(L,L')\le C(g,g|_L,g|_{L'})\sqrt{D(L,L')}. \end{aligned}$$

By the above notation, we mean that $\delta$ and *C* depend only on Riemannian bounds of *M*, *L*, and $L'$, e.g. the sectional curvature of the first and the $L^\infty$-norm of the second fundamental form of the two latter. The improvement in this note is that we get rid of the dependance of *C* on metric invariants of $L'$ at the price of an extra $\Vert \textrm{d}\psi \Vert$ term.

Note that the first author (Lemma 5 in [3]) partially improved (1.4) to

1.5 $$\begin{aligned} s(L;L')\le C(g,g|_L)\sqrt{\gamma (L,L')} \end{aligned}$$

whenever $$\gamma (L,L') < \delta = \delta (g,g|_{L})$$, where

1.6 $$\begin{aligned} s(L;L'){:}{=}\sup _{x\in L} d_M(x,L')=\sup _{x\in L}\inf _{y\in L'} d_M(x,y). \end{aligned}$$

Since $\delta _H(L,L')=\max \{s(L;L'),s(L';L)\}$, the left-hand side in (1.5) is in general smaller than the one in (1.4). It is through this inequality that Theorem 1.1 is proved (see Sect. 3).

### Relations to Joksimović and Seyfaddini’s inequality

Theorem 1.1 is a Lagrangian generalization of Joksimović and Seyfaddini’s aforementioned inequality (1.1) for Hamiltonian diffeomorphisms $\varphi$ on closed symplectic manifolds.

Note that their inequality directly implies

$$\begin{aligned} \delta _H(L,L')\le \inf _{\varphi (L)=L'} d_{C^0}(\mathbbm {1},\varphi )\le C\inf _{\varphi (L)=L'} \Big ( \sqrt{\gamma (\varphi )}\ \Vert \textrm{d}\varphi \Vert \Big ). \end{aligned}$$

However, in general, the inequality

$$\begin{aligned} \inf _{\varphi (L)=L'}\sqrt{\gamma (\varphi )}\ \Vert \textrm{d}\varphi \Vert&\ge \inf _{\varphi (L)=L'}\sqrt{\gamma (\varphi )}\,\cdot \!\!\inf _{\varphi (L)=L'} \Vert \textrm{d}\varphi \Vert \\&=\sqrt{\gamma (L,L')}\,\cdot \!\! \inf _{\varphi (L)=L'}\Vert \textrm{d}\varphi \Vert \end{aligned}$$

is strict. Therefore, our inequality gives a better bound in the Lagrangian case, even when $\varphi$ is a Hamiltonian diffeomorphism^1^.

One notable exception to this is when *L* is the diagonal in $M\times M$, and $L'$ is the graph of $\varphi$. Then, by work of the second author and Zapolsky [11, 12], we know that $\gamma (L,L')=\gamma (\varphi )$, so that equality follows. The constant we get here is, however, hard to compare to theirs.

On the other hand, we present below a different proof of a variant of (1.2), based on the method from [8] which gives a less natural, but more easily comparable, constant; see Sect. 4.1.

### Organization

After reviewing necessary preliminaries in Sect. 2, we prove Theorem 1.1 in Sect. 3. Finally, Sect. 4 presents the proofs of two inequalities similar to (1.2), the first one based on Joksimović and Seyfaddini’s method in Sect. 4.1, the second one involving the inverse norm of $\psi$ as mentioned in Remark 1.6; see Sect. 4.2.

## Preliminaries

We fix a symplectic manifold $(M,\omega )$ and consider different types of Lagrangian submanifolds. There are two well-known functions defined on the second homotopy group of *M* relative to a Lagrangian submanifold *L*, i.e. the symplectic area and the Maslov class of disks in *M* with boundary in *L*:

$$\begin{aligned} \omega _{L}: \pi _{2}(M,L) \rightarrow \mathbb {R}\quad \text{ and } \quad \mu _{L}: \pi _{2}(M,L) \rightarrow \mathbb {Z}. \end{aligned}$$

A Lagrangian submanifold *L* is called *weakly exact* if $\omega _{L}$ and $\mu _{L}$ vanish identically. Otherwise, *L* is called (positively) monotone whenever there exists a positive constant $\kappa _{L}>0$ such that $\omega _{L} = \kappa _{L} \cdot \mu _{L}$. In that case, $\kappa _{L}$ is called the monotonicity constant of *L*.

When *L* is monotone, we define its *minimal Maslov number* $N_{L}$ to be the positive generator of $\langle \mu _{L}, \pi _{2}(M,L) \rangle = N_{L}\,\mathbb {Z}$, and we require $N_{L} \ge 2$.

In what follows, we fix a Lagrangian as above and consider the set ${\mathcal {L}}^{\textrm{Ham}}(L)$ of all Lagrangian submanifolds which are Hamiltonian isotopic to *L*. We now recall how the two metrics on ${\mathcal {L}}^{\textrm{Ham}}(L)$ of interest in this note are defined.

### The Hofer–Chekanov distance

The Hofer norm was introduced by Hofer [7] on Hamiltonian diffeomorphism groups and extended as a distance to sets of the type ${\mathcal {L}}^{\textrm{Ham}}(L)$ by Chekanov [4].

First define the energy of a Hamiltonian function $H:[0,1]\times M\rightarrow \mathbb {R}$ as its $L^{(1,\infty )}$-norm:

2.1 $$\begin{aligned} \mathrm E(H) = \int _{0}^{1} \left( \max _{M}H_{t} - \min _{M}H_{t}\right) \, \textrm{d}t, \end{aligned}$$

where $H_t:=H(t,\cdot )$. Then, define the Hofer norm of a Hamiltonian diffeomorphism as

$$\begin{aligned} \Vert \varphi \Vert _{\textrm{Hof}} = \inf \left\{ \mathrm E(H) \,\big |\, \varphi _{H}^{1} = \varphi \right\} . \end{aligned}$$

Here, $\{\varphi _H^t\}_{t\in [0,1]}$ is the Hamiltonian flow of *H*, i.e. $\varphi _H^0=\mathbbm {1}_M$ and $\frac{\textrm{d}}{\textrm{d}t}\varphi _H^t=X_H^t\circ \varphi _H^t$, where $X_H^t$ is the unique time-dependent vector field of *M* such that $\iota (X_H^t)\omega =-\textrm{d}H_t$.

Hofer’s norm then yields a distance on ${\mathcal {L}}^{\textrm{Ham}}(L)$ by setting

$$\begin{aligned} d_{\textrm{Hof}}(L,L') = \inf \left\{ \Vert \varphi \Vert _{\textrm{Hof}} \,\big |\, \varphi (L) = L'\right\} = \inf \left\{ \mathrm E(H) \,\big |\, \varphi _{H}^{1}(L) = L' \right\} \end{aligned}$$

for any $$L' \in {\mathcal {L}}^{\textrm{Ham}}(L)$$.

#### Remark 2.1

As noted by Usher [16], replacing the Hofer energy $\mathrm E(H)$ in the above expression by the smaller quantity $\mathrm E_{L}(H)$, defined as in (2.1) but with oscillations taken only on *L* rather than on the whole ambient manifold *M*, yields the same distance.

### The Lagrangian spectral distance

This distance is based on the theory of spectral invariants initiated by Viterbo [17] via generating functions and adapted to Floer homology theories by Schwarz [15] and Oh [13] in the case of Hamiltonian diffeomorphism groups. The Lagrangian version which is of interest to us here was developed by the second author [11] in the weakly exact setting and by Zapolsky and the second author [12] in the monotone case—see also work by Fukaya et al. [6], which is based on more advanced techniques such as virtual fundamental cycles and Kuranishi structures.

*Lagrangian spectral invariants.* The Lagrangian spectral invariants $\ell (\alpha ;H)$ associated to *L* are defined for any nonzero quantum homology class $\alpha \in \textrm{QH}_{*}(L)$—see [1] for the construction of this homology. Since the Lagrangian spectral *distance* only relies on spectral invariants corresponding to the quantum fundamental class of *L*, we do not review the construction of the quantum homology of a Lagrangian, nor define spectral invariants in full generality. Instead, we assume that the quantum fundamental class of *L*, denoted [*L*], is nontrivial and only present the properties of $\ell _{+}:=\ell ([L], \,\cdot \,)$.

The function $\ell _{+}: C^{0}([0,1]\times M) \rightarrow {\mathbb {R}}$ satisfies the following properties.

1. Continuity. For any Hamiltonians *H* and *K*, we have that
  $$\begin{aligned} \int _{0}^{1}\min _{M}(K_{t}-H_{t}) \,dt \le \ell _{+}(K) - \ell _{+}(H) \le \int _{0}^{1}\max _{M} (K_{t}-H_{t}) \,dt . \end{aligned}$$
2. Triangle inequality. For all *H* and *K*, $\ell _{+}(H\sharp K) \le \ell _{+}(H) + \ell _{+}(K)$.
3. Lagrangian control. If $H_{t}|_{L} = c(t) \in {\mathbb {R}}$ (resp. $\le$, $\ge$) for all *t*, then
  $$\begin{aligned} \ell _{+}(H) = \int _{0}^{1}c(t) \, dt \qquad \text{(resp. } \le \text{, } \ge \text{). } \end{aligned}$$
4. Non-negativity. For all *H*, $\ell _{+}(H) + \ell _{+}({\overline{H}}) \ge 0$.
5. Homotopy invariance. If *H* is normalized, $\ell _{+}(H)$ only depends on the homotopy class relative to endpoints of the isotopy $\{\varphi _{H}^{t}\}_{t \in [0,1]}$, i.e. the class $[\{\varphi _{H}^{t}\}_{t \in [0,1]}] \in {\widetilde{\textrm{Ham}}}(M,\omega )$.
6. Symplectic invariance. For all *H* and all $\psi \in \textrm{Symp}(M,\omega )$, $\ell _{+}(H) = \ell '_{+}(H \circ \psi ^{-1})$.

Let us make a few comments about these properties and the notation used above.

- In Properties 2 and 4 respectively, $H\sharp K$ denotes the Hamiltonian function $H_{t}(x) + K\big ((\varphi _{H}^{t})^{-1}(x)\big )$ which generates the isotopy $\{\varphi _{H}^{t}\varphi _{K}^{t}\}_{t \in [0,1]}$, and ${\overline{H}}$ is the Hamiltonian function ${\overline{H}}_{t}(x) = - H_{t}\big ((\varphi _{H}^{t})^{-1}(x)\big )$ which generates $\big \{(\varphi _{H}^{t})^{-1}\big \}_{t \in [0,1]}$. Properties 1 to 4 are part of Theorem 3 in [12].
- Property 3 directly implies that for all *H*,
  $$\begin{aligned} \int _{0}^{1} \min _{L} H_{t} \,dt \le \ell _{+}(H) \le \int _{0}^{1} \max _{L} H_{t} \,dt . \end{aligned}$$
- In Property 5, the *normalization* refers to the fact that for all $t \in [0,1]$, $\int _{0}^{1} H_{t} \,\omega ^{n}=0$. This property appears as Proposition 4 in [12].
- Finally, concerning Property 6, note that any symplectomorphism $\psi$ induces an isomorphism $\psi _{*}: \textrm{QH}_{*}(L) \rightarrow \textrm{QH}_{*}(L')$ with $L' = \psi (L)$. The fundamental class of *L* is mapped to that of $L'$ through this action (up to possible multiplication by a unit of the coefficient field). The notation $\ell '_{+}$ denotes the Lagrangian spectral invariant associated with $L'$ (and its fundamental class). Now, Symplectic invariance only expresses the fact that spectral invariants agree with the action of $\psi$ by conjugation on the Hamiltonian diffeomorphism group: for any Hamiltonian function *H*, $\varphi _{H\circ \psi }^{t} = \psi ^{-1}\varphi _{H}^{t}\psi$. This result is part of Theorem 35 in [12].

*The Lagrangian spectral distance.* The properties of $\ell _{+}$ above show that not only $\ell _{+}$ defines a function on ${\widetilde{\textrm{Ham}}}(M,\omega )$ with similar properties (see Theorem 41 in [12]), but also a pseudodistance on ${\mathcal {L}}^{\textrm{Ham}}(L)$.

Indeed, following [10], first define the length of a Hamiltonian isotopy $\{\varphi _{H}^{t}\}_{t \in [0,1]}$ by $\gamma _{L}(H) = \ell _{+}(H)+\ell _{+}({\overline{H}})$, then take the infimum over all Hamiltonian isotopies which map *L* to $L'$:

$$\begin{aligned} \gamma (L,L') = \inf \{ \gamma _{L}(H) \,|\, \varphi _{H}^{1}(L) = L' \}. \end{aligned}$$

The Non-negativity property of $\ell _{+}$ ensures that $\gamma (L,\cdot )$ takes non-negative values. Symplectic invariance ensures that for any symplectomorphism $\psi$ and any Lagrangian $L' \in {\mathcal {L}}^{\textrm{Ham}}(L)$, $\gamma (L,L') = \gamma (\psi (L),\psi (L'))$. Combined with Triangle inequality, this shows that for all $L'$ and $L''$ in ${\mathcal {L}}^{\textrm{Ham}}(L)$,

$$\begin{aligned} \gamma (L,L'') \le \gamma (L,L') + \gamma (L',L''). \end{aligned}$$

Finally, note that if $L'=\varphi ^1_H(L)$, then $L=\varphi ^1_{\overline{H}}(L')$ and

$$\begin{aligned} \gamma _{L'}(\overline{H})&=\ell '_{+}(\overline{H})+\ell '_{+}(H)\\&=\ell _{+}(\overline{H}\circ \varphi ^1_H)+\ell _{+}(H\circ \varphi ^1_H)\\&=\ell _{+}(\overline{H\circ \varphi ^1_H})+\ell _{+}(H\circ \varphi ^1_H)\\&=\gamma _{L}(H\circ \varphi ^1_H), \end{aligned}$$

where the second line follows from Symplectic invariance, whilst the third one is a direct computation using the fact that $\varphi ^1_{H\circ \varphi ^1_H}=(\varphi ^1_H)^{-1}\varphi ^1_H\varphi ^1_H=\varphi ^1_H$. Since $\gamma (L',L)$ is defined by taking the infimum over all possible Hamiltonians whose diffeomorphism sends $L'$ to *L*, this implies symmetry for $\gamma$. This justifies the following definition.

#### Definition 2.2

Let *L* be a weakly exact Lagrangian or a monotone Lagrangian with $N_{L}\ge 2$ and nonzero quantum fundamental class. The *Lagrangian spectral distance* between $L_{0}$ and $L_{1} \in {\mathcal {L}}^{\textrm{Ham}}(L)$ is $\gamma (L_0,L_1)$.

The fact that this actually defines a *nondegenerate* distance is, as usual, the “hard” part. This was proven fairly simultaneously in [9] (via Poisson bracket invariants) and [10] (via energy-capacity inequality). This is also a consequence of the main result of the present note.

Finally, let us emphasize the fact that the Continuity property of $\ell _{+}$ obviously yields the well-known fact that

$$\begin{aligned} \hbox {for all}\,\, L' \in {\mathcal {L}}^{\textrm{Ham}}(L), \quad \gamma (L,L') \le d_{\textrm{Hof}} (L,L'). \end{aligned}$$

## Proof of Theorem 1.1

Fix a Lagrangian submanifold *L* which satisfies the assumptions of Theorem 1.1. Let $L' = \varphi _{H}^{1}(L) \in {\mathcal {L}}^{\textrm{Ham}}(L)$ for some Hamiltonian function *H*, and let $\psi \in \textrm{Symp}(M,\omega )$ be such that $L' = \psi (L)$. Notice that $\psi ^{-1}(L) \in {\mathcal {L}}^{\textrm{Ham}}(L)$ since the Hamiltonian function $H\circ \psi$ generates the isotopy $\{\psi ^{-1}\varphi _{H}^{t}\psi \}$ which maps $\psi ^{-1}(L)$ to *L* at time 1.

The Hausdorff distance between *L* and $L'$ is defined as $\delta _{H}(L,L') = \max \{s(L;L'),s(L';L)\}$, where *s*(*A*; *B*) is the supremum of the distance to *B* of a point in *A*; see formula (1.6).

From (1.5), i.e. Lemma 5 of [3], we get some constants $\delta =\delta (g,g|_L)>0$ and $C=C(g,g|_L)>0$ such that

$$\begin{aligned} s(L;\psi (L)) \le C\sqrt{\gamma (L,\psi (L))} \quad \text{ and } \quad s(L;\psi ^{-1}(L)) \le C\sqrt{\gamma (L,\psi ^{-1}(L))} \end{aligned}$$

whenever $\gamma (L,\psi (L))$ and $\gamma (L,\psi ^{-1}(L))$ are smaller than $\delta$.

Let $\ell (c)$ denote the length of a smooth path $c:[0,1]\rightarrow M$. Then,

$$\begin{aligned} s(\psi (L);L)&= \max _{y\in \psi (L)}\min _{\begin{array}{c} c(0)=y \\ c(1)\in L \end{array}} \ell (c) \\&= \max _{x\in L}\min _{\begin{array}{c} c(0)=x \\ c(1)\in \psi ^{-1}(L) \end{array}} \ell (\psi \circ c) \\&\le \Vert \textrm{d}\psi \Vert \max _{x\in L}\min _{\begin{array}{c} c(0)=x \\ c(1)\in \psi ^{-1}(L) \end{array}} \ell (c) \\&= \Vert \textrm{d}\psi \Vert \ s(L;\psi ^{-1}(L)). \end{aligned}$$

From this, we immediately get that

3.1 $$\begin{aligned} \delta _H(L,L')&\le \max \big \{s(L;\psi (L)),\Vert \textrm{d}\psi \Vert \ s(L;\psi ^{-1}(L))\big \} \nonumber \\&\le C\Vert \textrm{d}\psi \Vert \max \left\{ \sqrt{\gamma (L,\psi (L))},\sqrt{\gamma (L,\psi ^{-1}(L))}\right\} \end{aligned}$$

since $\Vert \textrm{d}\psi \Vert \ge 1$ for any symplectomorphism $\psi$. Indeed, a symplectic matrix must always have an eigenvalue with absolute value at least 1.

By Symplectic invariance, we know that $\gamma (L,\psi ^{-1}(L)) = \gamma (\psi (L),L)$, and (3.1) gives us the expected inequality (1.2):

$$\begin{aligned} \delta _{H}(L,L') \le C\Vert \textrm{d}\psi \Vert \sqrt{\gamma (L,L')} \end{aligned}$$

under the condition $\gamma (L,L')<\delta$.

To get rid of this condition when *M* is compact, we use Joksimović and Seyfaddini’s trick [8]: take *C* large enough so that

$$\begin{aligned} C\ge \frac{\textrm{Diam}(M)}{\sqrt{\delta }}. \end{aligned}$$

Then, if $\gamma (L,L')\ge \delta$, we trivially get

$$\begin{aligned} C\sqrt{\gamma (L,L')}\ \Vert \textrm{d}\psi \Vert \ge \textrm{Diam}(M)\ge \delta _H(L,L'), \end{aligned}$$

since $\Vert \textrm{d}\psi \Vert \ge 1$. Here, we have made use of the fact that the distance between two closed subsets of *M* is at most the diameter of *M*.

This ends the proof of Theorem 1.1. $\square$

## Alternative versions of inequality (1.2)

We conclude with two alternative versions of inequality (1.2): the first one is established by adapting to the Lagrangian setting Joksimović and Seyfaddini’s proof from [8], and the other one by using methods explored by Chassé and leading to inequality (1.4) from [2], rather than using directly inequality (1.5) from [3].

### Joksimović and Seyfaddini’s approach

We could have adapted Joksimović and Seyfaddini’s [8] proof of (1.1) to the Lagrangian context to get an analogous inequality. We give here the broad idea on how such an inequality is proven.

For each $x\in L$, take a Darboux chart $\psi _x:U_x\rightarrow \mathbb {R}^{2n}$ sending $L\cap U_x$ to $\mathbb {R}^n\times \{0\}$. Take also compact neighborhoods $K_x$ and $K'_x$ of *x* in *M* such that

$$\begin{aligned} K_x\subseteq \textrm{int}(K'_x)\subseteq K'_x\subseteq U_x. \end{aligned}$$

By compactness of *L*, we may take a finite subset $\{\psi _i\}_{1\le i\le k}$ of these charts, so that $\{\textrm{int}(K_i)\}_{1\le i\le k}$ still covers *L*. Then, setting

$$\begin{aligned} \varepsilon :=\min _{1\le i\le k} \min _{\begin{array}{c} x\in \partial K_i\\ x'\in \partial K'_i \end{array}} d(x,x') \quad \text{ and } \quad A:=\max _{1\le i\le k}\left\| \left. \textrm{d}\psi _i^{-1}\right| _{\psi _i(K'_i)}\right\| , \end{aligned}$$

we get the inclusion

$$\begin{aligned} \psi _i^{-1}(B^{2n}_r(\psi _i(x)))\subseteq B_{Ar}(x) \end{aligned}$$

for $x\in K_i$ and $r=2\sqrt{\frac{\gamma (L,L')}{\pi }}$ if $\gamma (L,L')<\delta =\frac{\pi \varepsilon ^2}{4A^2}$. Here, $B^{2n}$ denotes the Euclidean ball in $\mathbb {R}^{2n}$, whilst *B* is the metric ball in *M*. Note that $B^{2n}_r(\psi _i(x))$ is contained in the image of $\psi _i$, since $r<\frac{\varepsilon }{A}$. Indeed, $K'_i$ contains the metric ball $B_\varepsilon (x)$ by construction. But if $B^{2n}_{R}(\psi _i(x))$ is the largest Euclidean ball centered at $\psi _i(x)$ contained in $\psi _i(B_\varepsilon (x))$, then $\partial B^{2n}_{R}(\psi _i(x))\cap \partial \psi _i(B_\varepsilon (x))$ must contain some point *y* so that

$$\begin{aligned} R=|\psi _i(x)-y|\ge \Vert \left. \textrm{d}\psi _i^{-1}\right| _{\psi _i(K'_i)}\Vert ^{-1}d(x,\psi ^{-1}_i(y))\ge \frac{\varepsilon }{A} \end{aligned}$$

since the straight line from $\psi _i(x)$ to *y* is contained in $\psi _i(K'_i)$ by hypothesis. Thus, we have that $B^{2n}_{\varepsilon /A}(\psi _i(x))\subseteq \psi _i(B_\varepsilon (x))\subseteq \psi _i(K'_i)$.

If $Ar< d(x,L')$, the map $\psi _i^{-1}|_{B^{2n}_r(\psi _i(x))}$ would be a symplectic embedding of a ball of radius *r* with real part along *L* not crossing $L'$, so that

$$\begin{aligned} \gamma (L,L')\ge \frac{\pi }{2}r^2= 2\gamma (L,L') \end{aligned}$$

by the proof of Theorem E of [10], which is of course a contradiction. Therefore, we must have

$$\begin{aligned} d(x,L')\le 2A\sqrt{\frac{\gamma (L,L')}{\pi }}. \end{aligned}$$

By taking the maximum overall $x\in L$, this gives an inequality analogous to (1.5)—but with a constant depending on local charts on top of metric invariants of *M*. By applying this inequality to the case $L'=\psi (L)$ and $L'=\psi ^{-1}(L)$ for some symplectomorphism $\psi$ of *M*, we get (1.2) as in Sect. 3; the constant is now $C=\frac{2A}{\sqrt{\pi }}$.

### An inequality with the inverse norm

When *M* is compact, there is also an inequality with $\Vert \textrm{d}\psi ^{-1}\Vert$:

4.1 $$\begin{aligned} \delta _H(L,L')\le C\sqrt{\gamma (L,L')}\ \Vert \textrm{d}\psi ^{-1}\Vert ^2. \end{aligned}$$

The proof of (1.3) follows the scheme of the proof of (1.4) appearing in [2]. We thus recall the idea of said proof.

1. From the proof of Theorem E of [10], we know that there exist, for any $x\in L$ and any $x'\in L'$, *J*-holomorphic strips $u_x$ and $u_{x'}$ with boundary along *L* and $L'$—modulo arbitrarily small Hamiltonian perturbations—and passing through *x* and $x'$, respectively. Furthermore, their area is bounded from above by $2\gamma (L,L')$.
2. Using a version of the monotonicity lemma (see Proposition 2.1 in [2]), we get that
  $$\begin{aligned} \omega (u_x)\ge A(g,g|_L)r^2 \end{aligned}$$
  if the closed metric ball $B_r(x)$ does not intersect $L'$ and *r* is smaller than some $\delta =\delta (g,g|_L)>0$. There is an analogous result for $u_{x'}$ and $L'$. In particular, if $\gamma (L,L')$ is small enough, the inequality holds for all $r<d_M(x,L')$. Therefore, it holds for $r=d_M(x,L')$.
3. Taking the supremum over all $x\in L$ of the inequalities for *L*, we essentially get (1.5). Taking the supremum over all $x'\in L'$ of the inequalities for $L'$ gives an analogous inequality for the pair $(L',L)$. Taking the maximum of these two inequalities, we get (1.4).

We thus see that the dependence of *C* in (1.4) on metric invariants of $L'$ comes from the constant *A* in Step 2. Therefore, proving Theorem 1.1 reduces to proving the following proposition.

#### Proposition 4.1

There exist constants $\delta$ and *A* depending only on metric invariants of *M* and *L* with the following property.

Let $L' \in {\mathcal {L}}^{\textrm{Ham}}(L)$ and let $\psi$ be a symplectomorphism such that $\psi (L)=L'$. Let $\Sigma$ be a compact Riemann surface with boundary $\partial \Sigma$ with corners. Consider a nonconstant *J*-holomorphic curve $u':(\Sigma ,\partial \Sigma )\rightarrow (B_r(x'),\partial B_r(x')\cup L')$ for some $x'\in L'$ and $r\le \frac{\delta }{\Vert \textrm{d}\psi ^{-1}\Vert }$ such that $x'\in u'(\Sigma )$. Suppose that $u'$ sends the corners of $\Sigma$ to $\partial B_r(x')\cap L'$. Then,

$$\begin{aligned} \omega (u')\ge \frac{A}{\Vert \textrm{d}\psi ^{-1}\Vert ^2}r^2. \end{aligned}$$

Indeed, Proposition 2.1 of [2], Proposition 4.1, and Step (1) above yield

$$\begin{aligned} \min \left\{ \delta ,d_M(x,L')\right\}&\le C\sqrt{\gamma (L,L')} \end{aligned}$$

for all $x\in L$ and

$$\begin{aligned} \min \left\{ \frac{\delta }{\Vert \textrm{d}\psi ^{-1}\Vert },d_M(x',L)\right\}&\le C\sqrt{\gamma (L,L')}\Vert \textrm{d}\psi ^{-1}\Vert \end{aligned}$$

for all $x'\in L'$ (with $C=\frac{1}{\sqrt{2A}}$). In particular, if we suppose that $\gamma (L,L')<C^{-2}\delta ^2\Vert \textrm{d}\psi ^{-1}\Vert ^{-4}\le C^{-2}\delta ^2$, we get that

$$\begin{aligned} d_M(x,L')&\le C\sqrt{\gamma (L,L')} \end{aligned}$$

for all $x\in L$ and

$$\begin{aligned} d_M(x',L)&\le C\sqrt{\gamma (L,L')}\Vert \textrm{d}\psi ^{-1}\Vert \end{aligned}$$

for all $x'\in L'$. Taking the maximum over all *x* and all $x'$, we get $\delta _H(L,L')\le C\sqrt{\gamma (L,L')}\max \{1,\Vert \textrm{d}\psi ^{-1}\Vert \}$ as long as $\gamma (L,L')<C^{-2}\delta ^2\Vert \textrm{d}\psi ^{-1}\Vert ^{-4}$. This yields (4.1)—with the additional $\gamma$-smallness assumption—since $\Vert \textrm{d}\psi ^{-1}\Vert \ge 1$.

If $\gamma (L,L')\ge C^{-2}\delta ^2\Vert \textrm{d}\psi ^{-1}\Vert ^{-4}$, take $C'\ge C\delta ^{-1}\operatorname {Diam}(M)$, so that

$$\begin{aligned} C' \sqrt{\gamma (L,L')}\Vert \textrm{d}\psi ^{-1}\Vert ^2\ge C'C^{-1}\delta \ge \operatorname {Diam}(M)\ge \delta _{H}(L,L'), \end{aligned}$$

which gives the desired result.

Only Proposition 4.1 is thus now left to prove. To do so, we first need a new version of the isoperimetric inequality. Given an arc $\gamma ':([0,\pi ],\{0,\pi \})\rightarrow (M,L')$ whose image is contained in the metric ball $B_{\delta /\Vert \textrm{d}\psi ^{-1}\Vert }(x')$ for some $x'\in L'$, set $a(\gamma ')$ to be the symplectic area $\omega (u')$ of any map $u':\mathbb {D}\cap \{\operatorname {Im}z\ge 0\}\rightarrow M$ such that $u'(e^{i\theta })=\gamma '(\theta )$ and $u'(\mathbb {D}\cap \mathbb {R})\subseteq L'$. Here, $\mathbb {D}$ is the unit disk in $\mathbb {C}$.

First note that this definition is independent of the choice of extension $u'$. To see this, take

4.2 $$\begin{aligned} \delta =\min \left\{ \varepsilon ,\frac{\varepsilon }{2}r_\textrm{inj}(L),\frac{\varepsilon }{2}r_0,\frac{\pi }{4\sqrt{K_0}}\right\} \end{aligned}$$

if *L* is $\varepsilon$-tame (see [2] for the definition) and *M* has injectivity radius bounded away from zero by $r_0$ and sectional curvature with values in $[-K_0,K_0]$. Here, $r_\textrm{inj}(L)$ is the injectivity radius of *L* with the Riemannian metric induced by *M*. Then, for all $z\in \mathbb {D}\cap \{\operatorname {Im}z\ge 0\}$, we have that

$$\begin{aligned} d_M\left( \psi ^{-1}(u(z)),\psi ^{-1}(x')\right) \le \Vert \textrm{d}\psi ^{-1}\Vert \, d_M(u(z),x')\le \delta , \end{aligned}$$

i.e. $u:=\psi ^{-1}\circ u'$ has image in the metric ball $B_\delta (x)$ with $x:= \psi ^{-1}(x')\in L$. Take two extensions $u'_0$ and $u'_1$ of an arc $\gamma '$ as above, and denote $\alpha '_i:=u'_i|_\mathbb {R}$ and $\alpha _i:=\psi ^{-1}\circ \alpha '_i$. Then, $u_0\#\overline{u_1}$ is a disk whose boundary $\alpha _0\#\overline{\alpha _1}$ lies in *L*. Here, $\overline{f}(a+ib):=f(-a+ib)$ for any map $f:U\subseteq \mathbb {C}\rightarrow M$. But by $\varepsilon$-tameness of *L*, $\alpha _0\#\overline{\alpha _1}$ must be a loop in a metric ball of *L* (in the intrinsic metric) of radius $\frac{2\delta }{\varepsilon }\le r_\textrm{inj}(L)$, and must thus be contractible in the same ball. This nullhomotopy extends to a homotopy in a metric ball of *M* of radius $\frac{2\delta }{\varepsilon }$ of $u_0\#\overline{u_1}$ to a topological sphere. Since $\frac{2\delta }{\varepsilon }\le r_0$, this topological sphere must be itself contractible, so that

$$\begin{aligned} 0=\omega (u_0\#\overline{u_1})=\omega (u_0)-\omega (u_1)=\omega (u'_0)-\omega (u'_1), \end{aligned}$$

where the last inequality follows from the fact that $\psi$ is a symplectomorphism. In other words, $a(\gamma ')$ is indeed well defined.

We can now prove the following isoperimetric inequality.

#### Lemma 4.2

There exist constants $\delta$ and *B* depending only on metric invariants of *M* and *L* such that, for all arcs $\gamma ':([0,\pi ],\{0,\pi \})\rightarrow (M,L')$ with image in $B_{\delta /\Vert \textrm{d}\psi ^{-1}\Vert }(x')$ for some $x'\in L'$, we have that

$$\begin{aligned} a(\gamma ')\le B\Vert \textrm{d}\psi ^{-1}\Vert ^2\ell (\gamma ')^2. \end{aligned}$$

#### Proof

As noted above, we know that $\psi ^{-1}\circ \gamma$ has image in the metric ball $B_{\delta }(\psi ^{-1}(x'))$. Therefore, by said Lemma 2.1 of [2], we know that

$$\begin{aligned} a(\psi ^{-1}\circ \gamma )\le B(g,g|_L)\,\ell (\psi ^{-1}\circ \gamma )^2. \end{aligned}$$

However, $a(\psi ^{-1}\circ \gamma )=a(\gamma )$, since $\psi$ is a symplectomorphism, and $\ell (\psi ^{-1}\circ \gamma )\le \Vert \textrm{d}\psi ^{-1}\Vert \ell (\gamma )$, which give the desired inequality. $\square$

The proof of Proposition 4.1 then follows the same scheme as the proof of Proposition 2.1 of [2], except that we use Lemma 4.2 above—instead of Lemma 2.1 of [2]—to estimate the local action $a(\gamma ')$ for arcs $\gamma '$ with boundary on $L'$.

## Data Availability

Not applicable.

## Footnotes

1 Recall indeed that the symplectomorphism $\varphi$ needs not be Hamiltonian for Inequality (1.2) to hold.
